# Does Modified 5‐Item Frailty Index Correlate With Survival in Oropharyngeal Squamous Cell Carcinoma?

**DOI:** 10.1002/hed.70005

**Published:** 2025-08-08

**Authors:** Marta Chmielecka, Rhona Hurley, Mohd Afid Mohd Slim, Nichola Philp, Matthew Donachie, Jan Horackiewicz, Josh McGovern, Katrina Knight, Nicholas J. W. Rattray, Claire Paterson, Catriona M. Douglas

**Affiliations:** ^1^ School of Medicine, Dentistry and Nursing University of Glasgow Glasgow UK; ^2^ School of Cancer Sciences, Garscube Estate University of Glasgow Glasgow UK; ^3^ Glasgow Head and Neck Cancer (GLAHNC) Research Group Glasgow UK; ^4^ Department of Otolaryngology/Head and Neck Surgery Glasgow Royal Infirmary and Queen Elizabeth University Hospital Glasgow UK; ^5^ Department of Otolaryngology Forth Valley Royal Hospital, NHS Forth Valley Larbert UK; ^6^ Academic Department of General Surgery Glasgow Royal Infirmary Glasgow UK; ^7^ Strathclyde Institute of Pharmacy and Biomedical Sciences University of Strathclyde Glasgow UK; ^8^ Beatson West of Scotland Cancer Centre Glasgow UK

**Keywords:** frailty, HPV, mFI‐5, oropharyngeal cancer, survival

## Abstract

**Introduction:**

Oropharyngeal squamous cell cancer (OPSCC) is a common subtype of head and neck cancer. It is generally stratified into high, intermediate, and low risk prognostic groups on the basis of HPV (human papillomavirus) status and smoking history (Ang risk stratification). Frailty has been shown to have a negative survival effect in head and neck cancer. The effect of frailty on survival in OPSCC is unknown. The aim of this study was to explore overall survival (OS) and disease‐specific survival (DSS) in OPSCC based on the modified 5‐item frailty index (mFI‐5).

**Methods:**

This was a retrospective cohort study of patients diagnosed with OPSCC in the West of Scotland. The mFI‐5 is a validated score that categorizes patients according to their level of frailty. Univariate and multivariate survival analyses were performed.

**Results:**

One thousand and one patients with OPSCC diagnosed between 2012 and 2020 were identified. The median OS was shortened in both the 'Moderately Frail' (39 months) and 'Severely Frail' groups (11 months) compared with the 'Not Frail' group (82 months) (*p* < 0.001). DSS was shorter in the'Severely Frail' group (16 months) compared with the'Moderately Frail' group (66 months) (*p* < 0.001). However, after adjustment for other characteristics including HPV and PS (World Health Organization/Eastern Cooperative Oncology Group Performance Status), frailty was not significant on multivariate analysis.

**Conclusion:**

Survival in patients with OPSCC decreases significantly with increasing frailty as measured by the mFI‐5. However, PS and HPV status are better predictors of OS and DSS.

## 1 Introduction

The incidence of oropharyngeal squamous cell cancer (OPSCC) is rising in Western Countries, with Scotland experiencing particularly high rates and poor outcomes [1]. Key risk factors are HPV (human papillomavirus) infection and smoking history, with HPV‐positive OPSCC becoming increasingly common. Ang et al. [2] stratified oropharyngeal cancer into 3 categories—low, intermediate, and high risk of death—based on smoking pack years, tumor HPV status, and nodal or tumor size status, forming the foundation for current treatment strategies and clinical trials [3]. The average age of OPSCC diagnosis in the United Kingdom is 59.3 [4] but in OPSCC, it has been noted that advancing age is a poor prognostic marker [5] With frailty already being associated with a higher incidence of a range of cancers [6], it is of great importance to recognize frailty in the context of OPSCC and to understand the effect on patient outcomes.

Frailty, reflecting reduced reserve to cope with stressors, is associated with increased risk of disability, hospitalization, and death [7]. This impacts cancer outcomes, as frailty would reduce the likelihood of successful treatment due to this lack of reserve [8]. Frailty is more complex than age alone, as patients of any age can experience frailty, as demonstrated in studies which showed 11%–42% [9, 10] of young cancer survivors were frail. Many measures of frailty exist, including the Rockwood Clinical Frailty Scale [11] and the modified 11‐item frailty index (mFI‐11) [12].

The modified 5‐item frailty index (mFI‐5) is a concise version of the mFI‐11, using the subjective measure of “functional dependence,” that is, the need for assistance with activities of daily living alongside diagnoses of COPD, heart failure, diabetes, or hypertension to allow rapid assessment using information usually collected as part of patient workup. It has been demonstrated to be a good tool for predicting outcomes in various cancer types and in predicting outcomes in various surgical specialties [13, 14]. Patients with head and neck cancer have increased frailty compared with other solid tumor types [15], this has rarely been explored in specific anatomical subsites. A study from our center found that the majority of laryngeal squamous cell cancer patients were frail when measured by mFI‐5 [16]. The impact of frailty on survival in OPSCC is unknown and may be different due to the different risk factor profile.

This study evaluates the effect of frailty, assessed via mFI‐5, on overall survival (OS) and disease‐specific survival (DSS) in patients with OPSCC.

## 2 Materials and Methods

### 2.1 Patient Cohort

Patients diagnosed with OPSCC between January 2012 and June 2020 were identified from the West of Scotland Tumor Board database, allowing a minimum of 2‐year follow‐up. Inclusion criteria included histopathologically confirmed OPSCC discussed at the Tumor Board. Exclusions were non‐squamous cancers and cancers at other subsites.

Data was collected based on patient demographics (including deprivation scores calculated via Scottish Index of Multiple Deprivation [SIMD] quintile), tumor characteristics including HPV status, risk factors, Eastern Cooperative Oncology Group Performance Status (ECOG PS), treatment intention, and cause of death. SIMD is an area‐based measure of socio‐economic deprivation, based on an individual's postal code which encompasses seven domains (income, employment, education, health, access to services, crime and housing). Quintile 1 is the most deprived, and quintile 5 is the least deprived [17]. The date of the first presentation at the Tumor Board Meeting was used as the date of diagnosis.

### 2.2 Tumor Characteristics

Tumors were categorized by anatomical location (e.g., tonsil, soft palate, base of tongue, posterior pharyngeal wall, indeterminate location). TNM8 staging was retrospectively applied for cases before its 2017 adoption to ensure comparability. Advanced disease was defined as AJCC (American Joint Committee on Cancer) stage III/IV, and early disease as AJCC stage I/II.

### 2.3 Risk Factors

Alcohol use was categorized as current excess (> 14 units/week), previous excess, no consumption, occasional consumption, or unknown. Smoking was categorized as current, ex‐smoker, never smoked, or unknown. Patients with HPV‐positive disease who were current or ex‐smokers were classified as intermediate risk due to challenges in recording pack years. HPV status was determined using HPV‐PCR or p16 immunohistochemistry (> 70% p16 protein‐staining), as per national guidelines. HPV positivity was defined in this study as either p16 positivity or HPV PCR positivity. HPV positivity was defined in this study as either p16 positivity or HPV PCR positivity, as p16 testing is an internationally accepted surrogate marker of HPV status.

### 2.4 Treatment Intent

Treatment intent was recorded as curative or palliative. Recurrence data included local, regional, or distant recurrences.

### 2.5 mFI‐5 Calculation

Table 1 illustrates the method of calculating the mFI‐5 and assigning frailty categories. Patients with ECOG PS 0‐1 were considered to be functionally independent, and patients with ECOG PS of ≥ 2 were considered to be functionally dependent.

**TABLE 1:** mFI‐5 calculation.

Domain for mFI‐5 calculation	Points allocated
ECOG 0–1 (relatively independent)	0
ECOG 2–4 (relatively dependent)	1
COPD	1
Diabetes type 1 or 2	1
Heart failure	1
Hypertension requiring medication	1
mFI‐5 calculation	
$\frac{t\text{otal number of points}}{\text{total number of points available}}$	
mFI‐5 category	
< 0.2	Not Frail
≥ 0.2 to < 0.4	Moderately Frail
≥ 0.4	Severely Frail

As an example, a patient with PS score of 2 and a diagnosis of COPD would calculate as $\frac{1+1}{5}$ = 0.4 indicating severe frailty. The stratification into “Not frail”, “Moderately frail,” and “Severely frail” is in keeping with current literature [16, 18, 19].

### 2.6 Statistical Methodology

R studio version 2023.06.01 [20], with the survival, survminer, car, compareGroups, and ggplot2 packages, was used for statistical analysis [21, 22, 23, 24, 25]. Normality was tested with Shapiro–Wilk, and comparisons were made using unpaired *t*‐test or Wilcoxon test. Categorical data were analyzed with Chi‐squared tests or Fisher's exact tests. For contingency tables with small (< 5) expected counts where the Chi‐squared test assumptions were not met, Monte Carlo simulation was used to approximate *p*‐values [26]. Kaplan–Meier analysis was used for univariate survival (OS and DSS). OS was calculated from the date of diagnosis to death from any cause or last follow‐up; DSS was calculated from the same start date to death from OPSCC or last follow‐up. Patients without 5‐year follow‐up were censored at their last follow‐up date. Cox proportional hazard models were used for multivariate analysis, incorporating Ang risk stratification (HPV status, smoking, AJCC stage). Schoenfeld tests confirmed model assumptions (*p* > 0.05), and multicollinearity was acceptable (variance inflation factors < 2.5).

### 2.7 Ethics

This study obtained local board‐level approval for data handling. The Strengthening and Reporting of Observational Studies in Epidemiology (STROBE) guideline was followed in the design and reporting of this study. Full NHS Research Ethics Committee Review was not required, as assessed by the Medical Research Council and NHS Health Research Authority tool [27].

## 3 Results

### 3.1 Basic Demographics

One thousand and one patients were identified. Four hundred and eighty‐five patients (48.5%) had an mFI‐5 < 0.2, putting them into the “Not Frail” category, 350 (35%) were “Moderately Frail”, and 166 (16.5%) were classified as “Severely Frail.” Demographic differences by frailty category are shown in Table 2.

**TABLE 2:** Basic demographics and tumor characteristics.

Demographic	All (*n* = 1001)	Not Frail mFI‐5 = 0 (*n* = 485, 48.5%)	Moderately Frail mFI‐5 = 0.2 (*n* = 350, 35%)	Severely Frail mFI‐5 ≥ 0.4 (*n* = 166, 16.5%)	*p*
Median age	61 (55–68)	57 (52–63)	63 (57–71)	68 (62–74)	< 0.001^KW^
Age category					< 0.001^cs^
< 55	237 (23.7%)	180 (37.1%)	50 (14.3%)	7 (4.2%)	
55–65 years old	395 (39.5%)	204 (42.1%)	141 (40.3%)	50 (30.1%)	
> 65	369 (36.8%)	101 (20.8%)	159 (45.4%)	109 (65.7%)	
Sex					
Male	748 (74.7%)	369 (76.1%)	255 (72.9%)	124 (74.7%)	
Female	253 (25.3%)	116 (23.9%)	95 (27.1%)	42 (25.3%)	0.57^cs^
SIMD quintile					
1	395 (39.4%)	176 (36.3%)	142 (40.5%)	77 (46.4%)	
2	222 (22.2%)	100 (20.6%)	85 (24.3%)	37 (22.3%)	
3	157 (15.7%)	87 (17.9%)	45 (12.9%)	25 (15.1%)	
4	117 (11.7%)	56 (11.6%)	43 (12.3%)	18 (10.8%)	
5	110 (11%)	66 (13.6%)	35 (10%)	9 (5.4%)	0.03^cs^
ECOG PS					
0	541 (54.0%)	373 (76.9%)	144 (41.1%)	24 (14.5%)	
1	271 (27.1%)	112 (23.1%)	120 (34.3%)	39 (23.5%)	
2	111 (11.1%)	0 (0%)	53 (15.1%)	58 (34.9%)	
3	73 (7.3%)	0 (0%)	29 (8.3%)	44 (26.5%)	
4	5 (5%)	0 (0%)	4 (1.2%)	1 (0.6%)	< 0.001^cs^ ^mc^
AJCC stage (TNM 8)					
1	296 (29.6%)	174 (35.9%)	92 (26.3%)	30 (18.1%)	
2	166 (16.6%)	89 (18.3%)	57 (16.3%)	20 (12%)	
3	210 (21%)	97 (20%)	82 (23.4%)	31 (18.7%)	
4	319 (31.9%)	123 (25.4%)	116 (33.1%)	80 (48.2%)	
Missing/unstaged	10 (0.9%)	2 (0.4%)	3 (0.9%)	5 (3%)	< 0.001^cs^
Subsite					
Tonsil	417 (41.7%)	234 (48.2%)	124 (35.5%)	59 (35.5%)	
Base of tongue	284 (28.4%)	132 (27.2%)	106 (30.3%)	46 (27.7%)	
Soft palate	150 (15%)	67 (13.8%)	57 (16.3%)	26 (15.7%)	
Posterior pharyngeal wall	24 (2.4%)	10 (48.2%)	8 (2.3%)	6 (3.6%)	
Unspecified	126 (12.6%)	42 (8.7%)	55 (15.7%)	29 (17.5%)	0.002^cs^
Treatment intention					
Curative	728 (72.7%)	414 (85.4%)	243 (69.4%)	71 (42.8%)	
Palliative	273 (27.3%)	71 (14.6%)	107 (30.6%)	95 (57.2%)	< 0.001^cs^
HPV status					
Positive	498 (49.7%)	291 (60%)	155 (44.2%)	52 (31.3%)	
Negative	385 (38.5%)	153 (31.5%)	150 (42.9%)	82 (49.4%)	
Unknown	118 (11.8%)	41 (8.45%)	45 (12.9%)	32 (19.3%)	< 0.001^cs^
Ang risk category					
Low	181 (18.1%)	110 (22.7%)	56 (16%)	15 (9%)	
Intermediate	158 (15.8%)	89 (18.3%)	51 (14.6%)	18 (10.8%)	
High	278 (27.8%)	107 (22.1%)	110 (31.4%)	61 (36.7%)	
Unknown	384 (29.7%)	179 (36.9%)	133 (38%)	72 (43.4%)	< 0.001^cs^
Smoking status					
Current	488 (48.7%)	210 (43.3%)	185 (52.9%)	93 (56%)	
Ex	258 (25.8%)	129 (26.6%)	87 (24.9%)	42 (25.3%)	
Never	216 (21.6%)	131 (27%)	65 (18.6%)	20 (12%)	
Unknown	39 (3.9%)	15 (3.1%)	13 (3.7%)	11 (6%)	< 0.001^cs^
Alcohol consumption status					
Excess	261 (26.1%)	114 (23.5%)	106 (30.3%)	41 (24.7%)	
Previous excess	121 (12.1%)	49 (10.1%)	51 (14.6%)	21 (12.7%)	
Occasional	342 (34.2%)	185 (38.1%)	107 (30.6%)	50 (30.1%)	
None	186 (18.6%)	98 (20.2%)	58 (16.6%)	30 (18.1%)	
Unknown	91 (9.1%)	39 (8%)	28 (8%)	24 (14.5%)	0.01^cs^
Mean mFI‐5 score	0.14	0	0.2	0.475	< 0.001^KW^
Dependence at surgery (defined as PS ≥ 2)					
Yes	189 (18.9%)	0 (0%)	86 (24.6%)	103 (62%)	
No	812 (81.1%)	485 (100%)	264 (75.4%)	63 (38%)	< 0.001^FT^
COPD					
Yes	119 (11.9%)	0 (0%)	51 (14.6%)	68 (41%)	
No	882 (88.1%)	485 (100%)	299 (85.4%)	98 (59%)	< 0.001^FT^
Medicated hypertension					
Yes	308 (30.8%)	0 (0)	183 (52.3%)	125 (75.3%)	
No	693 (69.2%)	485 (100%)	167 (47.7%)	41 (24.7%)	< 0.001^FT^
Diabetes					
Yes	912 (91.1%)	0 (0%)	27 (7.7%)	62 (37.3%)	
No	89 (8.9%)	485 (100%)	323 (92.3%)	104 (62.7%)	< 0.001^FT^
Congestive heart failure					
Yes	23 (2.3%)	0 (0%)	5 (1.4%)	18 (10.8%)	
No	978 (97.7%)	485 (100%)	345 (98.6%)	148 (89.2%)	< 0.001^FT^

Abbreviations: CS, Chi squared test; CSMC, Chi‐squared test with Monte Carlo simulation; KW, Kruskal–Wallis test, FT, Fisher's exact test.

The overall median age within the cohort was 61, increasing across frailty groups (*p* < 0.001, Table 2). Increasing frailty is associated with increasing PS (*p* < 0.001) and current smoking status (*p* < 0.001). “Moderately Frail” patients have a higher prevalence of excessive alcohol consumption compared to those with severe frailty. Lower SIMD quintile, indicating greater socioeconomic deprivation, is associated with increasing frailty (*p* = 0.03).

### 3.2 Tumor Characteristics and Prognostic Groups

Tumor characteristics are shown in Table 2. Advanced stage disease was more common in patients with increasing frailty (*p* < 0.001). In terms of subsite, 41.7% of all tumors were located in the tonsil, but in “Not Frail” patients, this increased to 48.2%, suggesting a tonsillar site of origin is less common in frailer patients (*p* = 0.002).

In terms of HPV status, 49.7% of patients in the whole cohort had HPV‐positive disease. HPV‐positive disease has the highest prevalence in the “Not Frail” group (60%) but declines in the “Moderately Frail” (44.2%) and “Severely Frail” (31.3%) groups. Conversely, HPV‐negative disease increases from 31.5% in the “Not Frail” group to 49.4% in the “Severely Frail” group (*p* = 0.001). This suggests that HPV‐negative disease is associated with increasing frailty (*p* < 0.001). Similarly, when looking at Ang risk categories, the “Severely Frail” group has a higher proportion of high‐risk patients and a lower proportion of low‐risk patients compared with “Not Frail” patients (*p* < 0.001).

### 3.3 Treatment and Disease Progression Outcomes

The majority of patients (*n* = 728) had curative intent treatment, although this was less likely as frailty increased (*p* < 0.001, Table 2). Overall, for patients treated curatively (*n* = 728), 7.2% (53) had local recurrence, 11.7% (76) had regional recurrence, and 0.4% [3] patients had distal recurrence. There were no significant differences between frailty groups for local or regional recurrence by chi‐squared test.

### 3.4 Whole Cohort Survival Analysis

#### 3.4.1 Overall Survival

**FIGURE 1:**
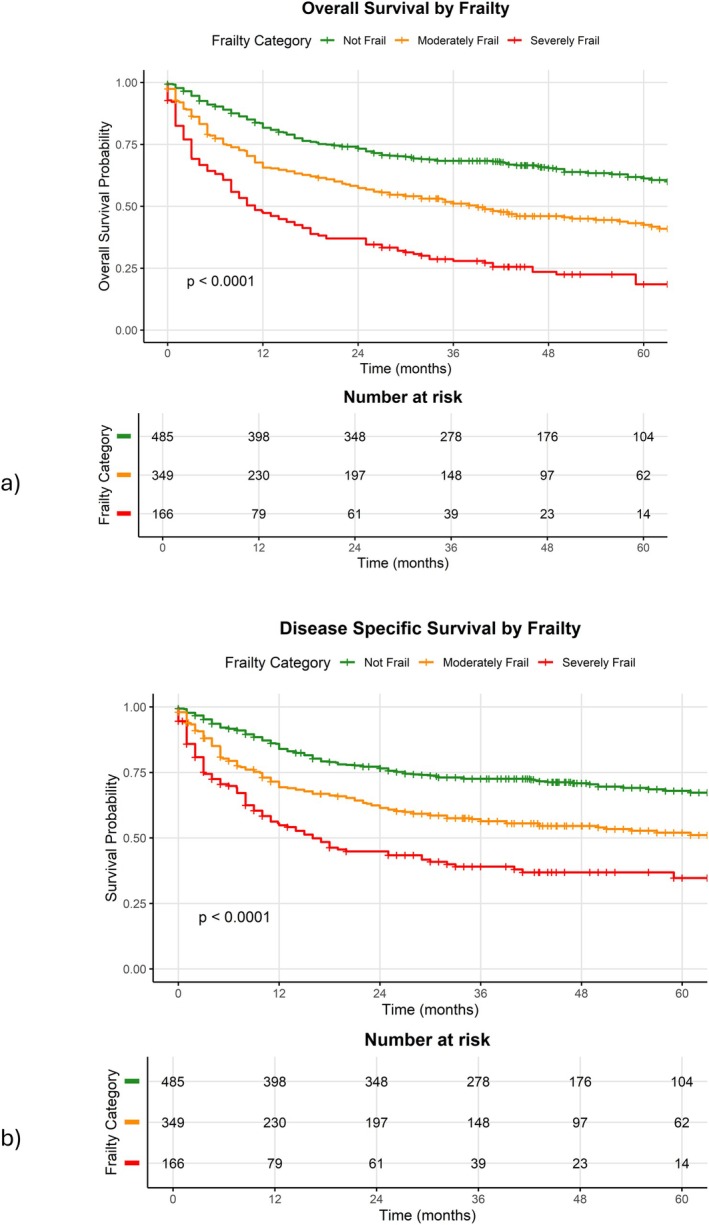
Kaplan–Meier curve for (a) OS and (b) DSS in the whole cohort. [Color figure can be viewed at wileyonlinelibrary.com]

The median OS for the “Not Frail” category (82 months, Table 3, Figure 1a) was longer compared to the “Moderately Frail” category and “Severely Frail” category (39 and 11 months, respectively, *p* < 0.0001). This trend was seen throughout the 5‐year follow‐up period. On multivariate analysis (Table 4a) frailty status was no longer statistically significant; however, PS, HPV status, intermediate Ang risk category, disease stage, and soft palate subsite remained statistically significant.

**TABLE 3:** OS and DSS outcomes by frailty status.

	Not Frail (mFI‐5 = 0)	Moderately Frail (mFI‐5 = 0.2)	Severely Frail (mFI‐5 ≥ 0.4)	*p* (log‐rank)
Overall survival				
Median survival (months)	82 (76–NA)	39 (28–57)	11 (8–17)	< 0.0001
1 year (%)	81.7 (78.3–85.2)	65.6 (60.8–70.8)	47.3 (40.3–55.6)	
2 year (%)	73.5 (69.6–77.5)	57.4 (52.2–62.9)	37.0 (30.3–45.1)	
3 year (%)	68.3 (64.28–72.7)	51.1 (46.0–56.7)	27.9 (21.8–35.8)	
4 year (%)	65.4 (61.1–70.0)	46.0 (40.9–51.8)	23.5 (17.6–31.5)	
5 year (%)	61.2 (56.5–66.4)	42.5 (37.1–48.7)	18.5 (12.7–27.0)	
Disease‐specific survival				
Median survival (months)	NA	66 (43–NA)	16 (11–30)	< 0.0001
1 year (%)	84.0 (80.7–87.3)	69.4 (64.7–74.5)	54.8 (47.5–63.4)	
2 year (%)	76.5 (72.8–80.5)	61.6 (56.5–67.1)	44.9 (37.5–53.6)	
3 year (%)	72.6 (68.6–76.8)	56.5 (51.4–62.2)	39 (31.8–48.0)	
4 year (%)	70.9 (66.7–75.2)	54.7 (49.4–60.5)	36.8 (29.5–46.0)	
5 year (%)	68.0 (63.4–72.8)	52.1 (46.6–58.3)	34.7 (27.0–44.6)	

**TABLE 4a:** Univariate and multivariate overall survival analysis of whole cohort.

Characteristic	Univariate analysis			Multivariate analysis		
	HR	95% CI	*p*	HR	95% CI	*p*
Age	1.04	1.03, 1.06	< 0.001	1.03	1.02, 1.04	< 0.001
Ang risk category						
Low	—	—		—	—	
Intermediate	2.24	1.36, 3.68	0.002	2.41	1.58, 3.70	< 0.001
High	4.45	2.86, 6.93	< 0.001	1.49	0.87, 2.54	0.15
Unknown	2.40	1.54, 3.74	< 0.001	1.41	0.90, 2.22	0.14
Disease stage						
Early	—	—		—	—	
Advanced	2.89	2.23, 3.74	< 0.001	2.53	2.03, 3.15	< 0.001
Subsite						
Base of tongue	—	—		—	—	
Posterior pharyngeal wall	3.02	1.61, 5.68	< 0.001	1.49	0.94, 2.36	0.094
Soft palate	1.05	0.73, 1.51	0.8	0.75	0.57, 0.99	0.041
Tonsil	0.62	0.46, 0.84	0.002	0.87	0.69, 1.09	0.2
Unspecified oropharynx	1.23	0.78, 1.94	0.4	1.07	0.80, 1.43	0.6
Frailty category						
Not Frail	—	—		—	—	
Moderately Frail	1.54	1.18, 2.02	0.002	0.93	0.73, 1.17	0.5
Severely Frail	2.42	1.68, 3.47	< 0.001	0.94	0.69, 1.28	0.7
PS						
PS 0	—	—		—	—	
PS 1	2.60	1.99, 3.39	< 0.001	1.71	1.36, 2.14	< 0.001
PS 2	3.94	2.59, 5.98	< 0.001	3.15	2.28, 4.34	< 0.001
PS 3	5.06	2.45, 10.4	< 0.001	6.09	4.19, 8.86	< 0.001
PS 4				3.57	1.25, 10.2	0.017
HPV status						
Positive	—	—		—	—	
Negative	2.85	2.19, 3.71	< 0.001	2.12	1.43, 3.12	< 0.001
Unknown	2.32	1.51, 3.57	< 0.001	1.96	1.34, 2.87	< 0.001

Abbreviations: CI, confidence interval; HR, hazard ratio.

#### 3.4.2 Disease‐Specific Survival

The median DSS for the “Not Frail” category could not be calculated as 50% of the patients were still alive at the time of censoring, but median survival in the Moderately Frail group was significantly longer than the “Severely Frail” group (66 months vs. 16 months, *p* < 0.0001, Table 3, Figure 1b). For multivariate analysis of DSS, the “Severely Frail” category was interestingly associated with better outcomes (*p* = 0.02, Table 4b) whereas unknown or negative HPV status, poor PS, intermediate Ang risk category, and advanced disease stage were associated with poor DSS.

**TABLE 4b:** Univariate and multivariate disease‐specific survival analysis of whole cohort.

	Univariate analysis			Multivariate analysis		
Characteristic	HR	95% CI	*p*	HR	95% CI	*p*
Age	1.04	1.03, 1.06	< 0.001	1.03	1.02, 1.04	< 0.001
Ang risk category						
Low	—	—		—	—	
Intermediate	2.24	1.36, 3.68	0.002	3.01	1.82, 4.99	< 0.001
High	4.45	2.86, 6.93	< 0.001	1.76	0.92, 3.35	0.086
Unknown	2.40	1.54, 3.74	< 0.001	1.54	0.89, 2.69	0.13
Disease stage						
Early	—	—		—	—	
Advanced	2.89	2.23, 3.74	< 0.001	3.44	2.62, 4.50	< 0.001
Subsite						
Base of tongue	—	—		—	—	
Posterior pharyngeal wall	3.02	1.61, 5.68	< 0.001	1.19	0.68, 2.07	0.5
Soft palate	1.05	0.73, 1.51	0.8	0.64	0.46, 0.89	0.008
Tonsil	0.62	0.46, 0.84	0.002	0.87	0.67, 1.13	0.3
Unspecified oropharynx	1.23	0.78, 1.94	0.4	1.04	0.76, 1.42	0.8
Frailty category						
Not Frail	—	—		—	—	
Moderately Frail	1.54	1.18, 2.02	0.002	0.84	0.64, 1.09	0.2
Severely Frail	2.42	1.68, 3.47	< 0.001	0.66	0.46, 0.95	0.025
Performance status						
PS 0	—	—		—	—	
PS 1	2.60	1.99, 3.39	< 0.001	1.59	1.22, 2.06	< 0.001
PS 2	3.94	2.59, 5.98	< 0.001	4.08	2.84, 5.87	< 0.001
PS 3	5.06	2.45, 10.4	< 0.001	7.06	4.59, 10.9	< 0.001
PS 4				2.17	0.51, 9.24	0.3
HPV status						
Positive	—	—		—	—	
Negative	2.85	2.19, 3.71	< 0.001	2.11	1.33, 3.35	0.002
Unknown	2.32	1.51, 3.57	< 0.001	1.93	1.22, 3.04	0.005

### 3.5 Survival Analysis by Treatment Intent and Frailty

**FIGURE 2:**
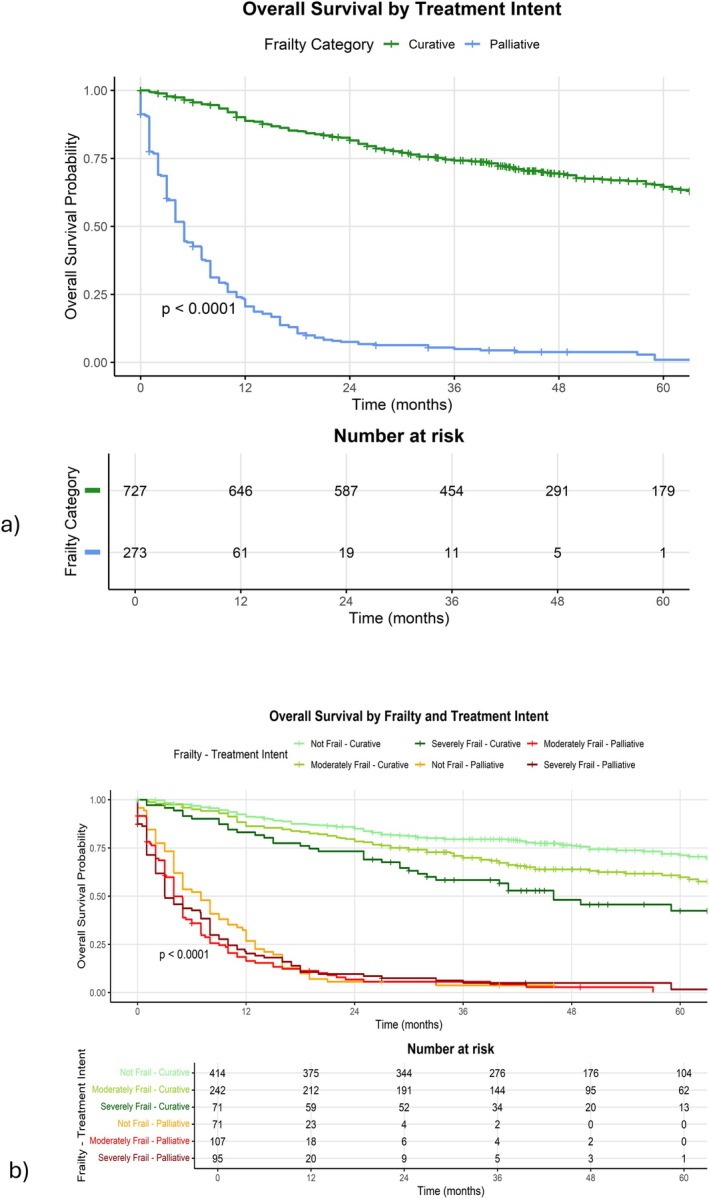
(a) Kaplan–Meier curve for palliative versus curative survival and (b) Kaplan–Meier curve for frailty category and treatment intention. [Color figure can be viewed at wileyonlinelibrary.com]

The median overall survival for the “Curative” treatment intention group (100 months, Figure 2) was longer compared to the “Palliative” group (5 months, *p* < 0.0001).

The median survival between the “Not Frail + Palliative” group (7 months, Figure 2b) and “Severely Frail + Palliative” (3 months) groups was not statistically significant (Log‐rank *p* = 0.33).

However, when looking at patients treated with curative intent, there is a statistically significant difference between median survival of “Not Frail + Curative” (NA, Figure 2b) and “Moderately Frail + Curative” (79 months) (Log‐rank *p* = 0.002). Similarly, the difference between “Moderately Frail + Curative” and “Severely Frail + Curative” (46 months) was statistically significant (Log rank *p* = 0.02).

### 3.6 Curative Cohort Survival Analysis

#### 3.6.1 Overall Survival

**FIGURE 3:**
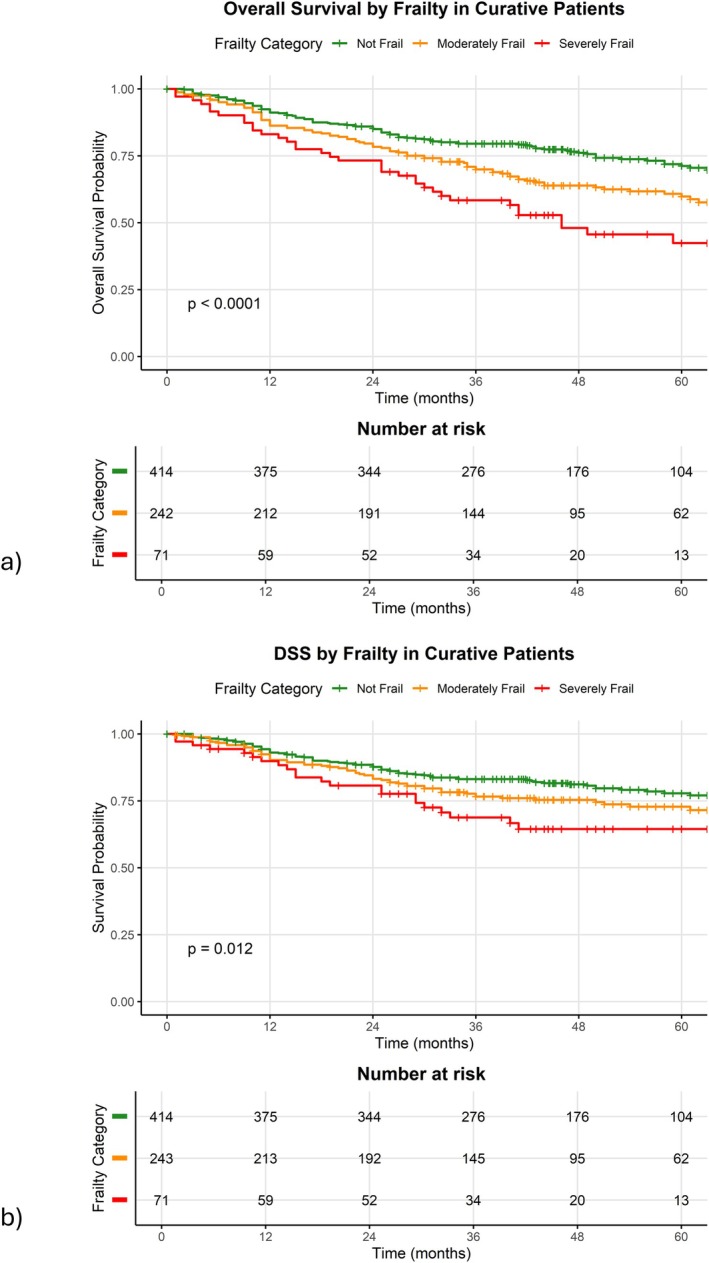
(a) Overall survival by frailty in curatively treated patients and (b) disease‐specific survival by frailty in curatively treated patients. [Color figure can be viewed at wileyonlinelibrary.com]

In the curative cohort, median survival could not be calculated in patients in the Not Frail category as 50% of the patients were still alive at the time of censoring. Median survival was 79 months in the “Moderately Frail” group and 46 months in the “Severely Frail” group (*p* < 0.001, Figure 3).

Multivariate survival analysis was repeated for the curative cohort. For OS (Table 5a), frailty status was not independently associated with survival, but age, HPV status, PS, intermediate Ang Risk, and stage were associated with poorer OS. Posterior pharyngeal wall disease was associated with worse OS.

**TABLE 5a:** Univariate and multivariate overall survival analysis in curatively treated patients.

Characteristic	Univariate analysis			Multivariate analysis		
	HR	95% CI	*p*	HR	95% CI	*p*
Age	1.04	1.03, 1.06	< 0.001	1.02	1.00, 1.04	0.020
Ang risk category						
Low	—	—		—	—	
Intermediate	2.24	1.36, 3.68	0.002	2.02	1.22, 3.35	0.006
High	4.45	2.86, 6.93	< 0.001	1.35	0.68, 2.66	0.4
Unknown	2.40	1.54, 3.74	< 0.001	1.26	0.74, 2.14	0.4
Disease stage						
Early	—	—		—	—	
Advanced	2.89	2.23, 3.74	< 0.001	2.21	1.67, 2.93	< 0.001
Subsite						
Base of tongue	—	—		—	—	
Posterior pharyngeal wall	3.02	1.61, 5.68	< 0.001	2.05	1.04, 4.02	0.037
Soft palate	1.05	0.73, 1.51	0.8	0.71	0.48, 1.04	0.082
Tonsil	0.62	0.46, 0.84	0.002	0.78	0.57, 1.05	0.11
Unspecified oropharynx	1.23	0.78, 1.94	0.4	0.95	0.59, 1.53	0.8
Frailty category						
Not Frail	—	—		—	—	
Moderately Frail	1.54	1.18, 2.02	0.002	1.05	0.78, 1.41	0.7
Severely Frail	2.42	1.68, 3.47	< 0.001	1.12	0.71, 1.76	0.6
PS						
PS 0	—	—		—	—	
PS 1	2.60	1.99, 3.39	< 0.001	1.65	1.23, 2.20	< 0.001
PS 2	3.94	2.59, 5.98	< 0.001	2.57	1.54, 4.26	< 0.001
PS 3	5.06	2.45, 10.4	< 0.001	6.14	2.77, 13.6	< 0.001
HPV status						
Positive	—	—		—	—	
Negative	2.85	2.19, 3.71	< 0.001	1.98	1.17, 3.36	0.011
Unknown	2.32	1.51, 3.57	< 0.001	1.60	0.92, 2.78	0.10

Abbreviations: CI, confidence interval; HR, hazard ratio.

#### 3.6.2 Disease‐Specific Survival

For DSS (Figure 3b), median survival could not be calculated in any frailty category as 50% of the patients were still alive at the time of censoring, but “Severely Frail” patients had worse DSS (*p* < 0.001, Figure 1b, Table 5b). In multivariate survival analysis, frailty status was not independently associated with survival, but PS, intermediate Ang Risk, and stage were associated with poorer DSS.

**TABLE 5b:** Univariate and multivariate disease‐specific survival analysis in curatively treated patients.

Characteristic	Univariate analysis			Multivariate analysis		
	HR	95% CI	*p*	HR	95% CI	*p*
Age	1.03	1.01, 1.05	< 0.001	1.01	0.99, 1.03	0.3
Ang risk category						
Low	—	—		—	—	
Intermediate	3.07	1.67, 5.66	< 0.001	2.66	1.43, 4.94	0.002
High	5.61	3.20, 9.83	< 0.001	1.94	0.79, 4.75	0.15
Unknown	2.26	1.27, 4.03	0.006	1.30	0.65, 2.60	0.5
Disease stage						
Early	—	—		—	—	
Advanced	3.85	2.76, 5.37	< 0.001	2.92	2.04, 4.17	< 0.001
Subsite						
Base of tongue	—	—		—	—	
Posterior pharyngeal wall	2.35	1.01, 5.46	0.047	1.91	0.78, 4.66	0.2
Soft palate	0.93	0.59, 1.47	0.7	0.63	0.39, 1.01	0.055
Tonsil	0.54	0.37, 0.79	0.001	0.72	0.50, 1.06	0.10
Unspecified oropharynx	1.42	0.86, 2.35	0.2	0.94	0.55, 1.61	0.8
Frailty category						
Not Frail	—	—		—	—	
Moderately Frail	1.35	0.97, 1.88	0.079	0.88	0.61, 1.28	0.5
Severely Frail	1.93	1.21, 3.07	0.005	0.72	0.40, 1.31	0.3
PS						
PS 0	—	—		—	—	
PS 1	2.06	1.47, 2.90	< 0.001	1.35	0.94, 1.95	0.11
PS 2	4.82	3.07, 7.58	< 0.001	3.91	2.16, 7.05	< 0.001
PS 3	4.11	1.50, 11.2	0.006	6.34	2.15, 18.7	< 0.001
HPV status						
Positive	—	—		—	—	
Negative	2.93	2.12, 4.05	< 0.001	1.77	0.87, 3.59	0.11
Unknown	2.19	1.25, 3.84	0.006	1.58	0.76, 3.29	0.2

Abbreviations: CI, confidence interval; HR, hazard ratio.

### 3.7 Frailty and HPV Status

Frailty and HPV status were combined in those who had known HPV status (*n* = 675) to assess the difference between HPV‐positive and HPV‐negative tumors.

#### 3.7.1 Overall Survival

Median survival could not be calculated in the HPV positive group who were in the “Not Frail” and “Moderately Frail” groups as 50% of the patients were still alive at the time of censoring. Median survival was 73 months in the HPV positive “Severely Frail” group. Median survival was lower in the HPV negative group, with “Not Frail” patients having median survival of 67 months, “Moderately Frail” patients having median survival of 51 months, and “Severely Frail” patients having a median survival of 30 months.

**FIGURE 4:**
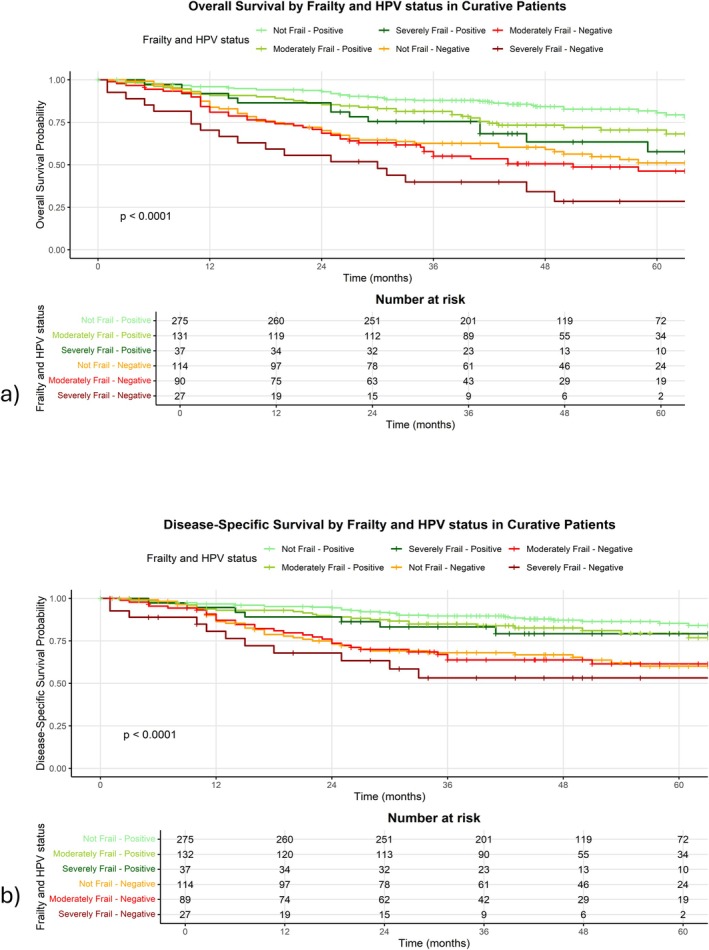
(a) Overall survival analysis by frailty and HPV status and (b) Disease‐specific survival by frailty and HPV status, both in curatively treated patients. [Color figure can be viewed at wileyonlinelibrary.com]

Comparison of overall survival by frailty and HPV status in curative patients is shown in Figure 4 and Table 6, which demonstrates a worsening by frailty category, but this is stratified by HPV status, with HPV negative patients experiencing worse survival than HPV positive, but increasing frailty demonstrates worse survival in both categories.

**TABLE 6:** Univariate survival analysis by frailty and HPV status.

Category	HR	*p*	Lower 95% CI	Upper 95% CI
Overall survival				
Reference: Not Frail—HPV positive				
Moderately Frail—HPV positive	1.64	0.03	1.06	2.54
Severely Frail—HPV positive	2.52	0.001	1.43	4.46
Not Frail—Negative	3.16	< 0.001	2.14	4.68
Moderately Frail—Negative	3.90	< 0.001	2.63	5.80
Severely Frail—Negative	6.55	< 0.001	3.84	11.19
Disease‐specific survival				
Reference: Not Frail—HPV positive				
Moderately Frail—positive	1.51	0.12	0.90	2.56
Severely Frail—positive	1.84	0.12	0.85	3.98
Not Frail—negative	3.35	< 0.001	2.12	5.30
Moderately Frail—negative	3.51	< 0.001	2.17	5.69
Severely Frail—negative	5.01	< 0.001	2.54	9.90

#### 3.7.2 Disease‐Specific Survival

Median survival could not be calculated in any category as 50% of patients were still alive at the time of censoring. Comparison of DSS by frailty and HPV status in the curative cohort is shown in Figure 4b and Table 6, which demonstrates a worsening by frailty category, but this is stratified by HPV status, with all HPV negative patients experiencing worse survival than HPV positive, but increasing frailty demonstrates worse survival in both categories.

## 4 Discussion

The impact of frailty in OPSCC is unknown. Previous research in our centre, examining laryngeal SCC, found that 75% of patients are “Moderately Frail” or “Severely Frail” as measured by the mFI‐5 [16]. OPSCC, however, is divided into HPV‐positive and HPV‐negative disease, which are clinically distinct, with different staging and outcomes. This study aimed to evaluate the impact of frailty on survival in OPSCC and to assess whether this impact differs between HPV‐positive and HPV‐negative disease.

This is a large cohort of 1001 patients with OPSCC. Most patients (74.7%) were male, and the most common subsite was the tonsil (41.7%), consistent with a recent French cohort [28]. HPV‐positive disease was present in 49.7% of patients in our cohort, aligning with United Kingdom (51.8%) [4] and French data (increase from 43% to 57.3% from 2011 to 2021) [28] but lower than the United States (71.7%) [29]. Smoking was prevalent in our cohort, with 48.7% current or 25.8% ex‐smokers (25.8%). Most patients were treated with curative intent (72.7%), consistent with other studies [28].

Frailty prevalence, as defined by mFI‐5, was 51.5% in this cohort, and of this, 35% were classified as moderately frail, and 16.5% as severely frail. Patients with HPV‐negative disease were more likely to be frail (60%) compared to patients with HPV‐positive disease (41.5%, *p* < 0.001). This rate is higher than a report of 138 patients with OPSCC which described a frailty rate of 41% using mFI‐5 [30], although patients in this study all underwent TORS (Transoral robotic surgery) so were treated curatively, and 83% had HPV‐positive disease. Jain et al. have demonstrated that frailty as measured by mFI‐5 was associated with increased risk of post‐operative complications and mortality following TORS surgery for head and neck cancers, even when controlling for age [31]. Median age increases with frailty increases from 57 (Not Frail) to 68 (Severely Frail) (*p* < 0.001), confirming an association between frailty and age. Current cigarette smoking was more common in patients who were “Moderately” or “Severely Frail,” which is unsurprising as smoking is a predictor of increasing frailty [32]. “Severely Frail” patients tended to present with more advanced disease (*p* < 0.001) and often had tumors originating from non‐tonsillar subsites (*p* = 0.002). Advanced disease alone could impact PS, making it difficult to determine whether frailty is a result of advanced disease or is linked to the likelihood of presenting with advanced disease. Most patients in the “Not Frail” (85.4%) and “Moderately Frail” (69.4%) groups received curative intent treatment. Curative treatment intention was less likely in the “Severely Frail” group (42.8%), again likely due to multimorbidity and poor PS.

Previously, frailty has often been examined in heterogenous cohorts of patients with head and neck squamous cell cancer (HNSCC) with limited focus on subsite specific disease. Frailty has been correlated with worse outcomes across treatment modalities in HNSCC, with non‐surgical outcomes being equivalent or worse than those treated surgically [33]. Nieman et al. were able to demonstrate that frailty was associated with mortality in a large cohort of patients with HNSCC of all subsites undergoing surgical treatment [34]. Bakas et al. examined frailty in patients with HNSCC who were < 70 years of age and found significantly higher mortality at 1 year in patients who were frail [35]. There is limited study of frailty in OPSCC specifically, which may be of importance given the different profile of HPV‐driven OPSCC. Our study adds to the literature by showing that increasing frailty is associated with poorer OS and DSS in all OPSCC patients and those treated with curative intent, although this effect did not persist in multivariate analysis.

The links between deprivation and head and neck cancer are well established. Over half of the cohort lived in the two most deprived SIMD quintiles. 39.4% of patients were in the most deprived group SIMD quintile 1. Severe frailty was associated with deprivation (*p* < 0.001). SIMD quintile 1 had the highest proportion of “Severely frail” patients (46.4%) compared to only 5.4% of patients in this group in SIMD quintile 5. Frailty has been shown to be associated with socioeconomic deprivation in the United Kingdom [36] and the United States [37]. In Scotland, one nation of the United Kingdom, the areas with the highest levels of deprivation have the highest rates of OPSCC [1], which is likely to affect the prevalence of frailty in our population. There is limited data regarding deprivation and frailty in HNSCC; however, Bakas et al. did measure the level of education as a surrogate marker of deprivation and found that frail patients had a significantly lower level of formal education compared with non‐frail patients [35]. This is in keeping with the higher prevalence of frailty in patients living in an area of higher deprivation in our cohort.

Although frailty was not significant on multivariate analysis when examining survival in the whole or curative cohort; Ang risk stratification, HPV, and PS remain independent predictors of survival on multivariate analysis. This raises the question of whether mFI‐5 scores confer any advantage over currently used metrics such as PS. Although PS has been criticized for being a subjective measure, increasing PS consistently is associated with worse survival outcomes, indicating this is generally used appropriately. High‐risk OPSCC patients, as defined by Ang [2] unsurprisingly had worse OS and DSS. Patients with HPV‐driven disease have better OS and DSS than patients with HPV‐negative disease, regardless of frailty status. Within each disease subtype, there is worsening survival as frailty increases. It was interesting to note the relatively good outcomes for patients with HPV‐driven disease, even in the “Severely frail” category, with the caveat that these are likely to be highly selected cases. When tumor boards are making treatment decisions in patients with HPV‐driven OPSCC, it may be that “Severe frailty” as defined here does not necessarily indicate that these patients should not receive radical treatment. A potential area for further research is trying to identify which patients with HPV‐positive tumors may benefit from treatment despite frailty, allowing opportunities for optimizing and supporting them through treatment. This underpins the importance of looking in detail at HNSCC by differing subsites, given the different disease course between HPV‐positive and HPV‐negative disease.

A PS score of 0 was most common among all patients (54%). A PS ≥ 2 occurred in 24.6% of “Moderately Frail” patients and 62.6% of “Severely Frail” patients, as opposed to 0% of the “Not Frail” group. This indicated that increasing PS is significantly associated with increasing frailty (*p* < 0.001), and PS, although subjective, is likely correctly applied to patients in the MDT discussion and is a strong predictor of mortality, outperforming mFI‐5 in multivariate analysis. A recent study has found it is safe to select patients with HNSCC aged over 70 years for radical radiotherapy using assessment of PS and comorbidities without the need for frailty assessment and demonstrated that PS is the strongest predictor of survival in patients undergoing radical radiotherapy [38]. This supports our own observations, both in this cohort and in laryngeal cancer [16].

It may be that the mFI‐5 is not the optimal way to assess frailty in patients with OPSCC. We report results of 28% of patients in the “Severely frail” group as having PS of 0–1, demonstrating its limitations. It does not account for BMI or weight loss, yet malnutrition is associated with mortality in HNSCC [39]. Objective radiological assessment of sarcopenia [40, 41] may contribute here, while serum metabolomic markers [42], TNF‐alpha, IL‐6 [43] and neutrophil–lymphocyte ratio may have a role [44, 45]. These are promising avenues for future research that could lead to complementary pharmacological interventions for frailty.

The mFI‐5 tool may be useful in guiding prehabilitation and optimization strategies by screening patients for frailty. Our study did not look at individual treatment regimens in detail. As Jain et al. have indicated, increasing mFI‐5 being associated with poorer outcomes after TORS may highlight a potential role for frailty screening using the mFI‐5 [31]. This could involve early referral to care of the elderly teams. Frail patients may benefit from a comprehensive frailty assessment using the experience of all members of the multi‐disciplinary team to advise the patient better in decision making regarding the risks of surgery and the anesthetic, side effects of chemoradiotherapy, and discharge planning [46]. In palliative settings, early frailty detection could enhance symptom management and align care with patient preferences [47].

### 4.1 Strengths and Limitations

This is the first study to examine the role of mFI‐5 in assessing frailty and its impact on survival in patients with OPSCC. This large cohort of patients enhances generalizability of the findings. However, the retrospective nature of data collection introduces limitations, with missing data on smoking history and HPV status. Consequently, Ang risk could not be calculated in 29.7% of patients. Patients with HPV‐positive disease who were current or ex‐smokers were classified as intermediate risk. This approach assumes most patients who smoke in the United Kingdom begin prior to age 18 [48] and smoke an average of 9 cigarettes daily [49]. While the median age of this cohort supports the assumption of a 10 pack‐year history in current or ex‐smokers, some over‐estimation is possible. This study did not evaluate the impact of frailty on specific treatments. This should be a focus of future research to determine the impact of frailty by different OPSCC treatments, and to determine if frailty is related to treatment morbidity.

## 5 Conclusion

Frailty is common in OPSCC, particularly among patients with HPV‐negative disease. It is essential for surgeons and oncologists to understand the implications of frailty when managing these patients. While frailty, as measured by the mFI‐5, was associated with poorer OS and DSS in univariate analysis, it was not an independent predictor of survival in multivariate models. In contrast, HPV status and performance status remained significant predictors. Further research is needed to clarify the role of frailty in OPSCC outcomes and to guide treatment decisions for frail patients.

## Author Contributions

M.C. – data curation, analysis, visualization, writing (original draft, editing). R.H. – conceptualization, data curation, analysis, methodology, visualization, writing (original draft, editing). M.A.M.S. – data curation, analysis, visualization, methodology, writing – review and editing. N.P. – data collection, writing – review and editing. M.D. – data collection, writing – review and editing. J.H. – data collection, data curation, writing – review and editing. J.M. – conceptualization, supervision, writing – review and editing. K.K. – conceptualization, supervision, writing – review and editing. N.J.W.R. – supervision, writing – review and editing. C.P. – supervision, writing – review and editing. C.M.D. – conceptualization, data curation, supervision, writing – review and editing.

## Data Availability

The data that support the findings of this study are available on request from the corresponding author. The data are not publicly available due to privacy or ethical restrictions.
